# Impact of Biologic Treatment of Crohn’s Disease on the Rate of Surgeries and Other Healthcare Resources: An Analysis of a Nationwide Database From Poland

**DOI:** 10.3389/fphar.2018.00621

**Published:** 2018-06-11

**Authors:** Przemysław Holko, Paweł Kawalec, Andrzej Pilc

**Affiliations:** ^1^Institute of Public Health, Drug Management Department, Jagiellonian University Medical College, Kraków, Poland; ^2^Institute of Pharmacology, Department of Neurobiology, Polish Academy of Sciences, Kraków, Poland

**Keywords:** Crohn disease, biologic treatment, surgery, hospitalization, infliximab, adalimumab, TNF antagonists

## Abstract

**Background:** There is conflicting evidence on the impact of biologic treatment on the rate of complications and surgeries in Crohn’s disease (CD). We aimed to assess real-world consequences of biologic treatment of CD.

**Methods:** All adult patients with CD treated with infliximab and adalimumab in the years 2012–2014 were identified from the database of the National Health Fund in Poland. Mixed models were used to assess the impact of biologics on medical resource utilization by comparing the periods before and after the first use of biologics (pre-index vs. post-index). The additional analyses including quintile of total exposure to biologic treatment were performed.

**Results:** Data on 1393 patients (age, 31.9 years; males, 52.6%) were analyzed over a median of 1064 days (range: 71, 1148). During the post-index period, patients received from one to four treatments with biologic agents (maximum allowed period of 12 months per treatment). We observed a reduction in the rates of surgeries (by 27%, *p* = 0.001), hospitalizations for CD excluding surgical procedures (by 45%, *p* < 0.001), as well as consumption of antibiotics (by 31%, *p* < 0.001) and steroids (by 35%, *p* < 0.001) in the post-index compared with the pre-index period. The reduction in the rate of surgeries, hospitalizations for CD, and steroid intake increased with the increase of exposure to biologic agents.

**Conclusion:** Biologic treatment changed the management patterns by lowering the rate of surgeries and other healthcare resources related to complications or worsening of CD. The reduction in the resource utilization was dependent on the level of exposure to treatment, suggesting that limitation of the treatment period itself may be inadequate.

## Introduction

Crohn’s disease (CD) is a chronic, relapsing inflammatory disease affecting the gastrointestinal tract, with intestinal and extraintestinal complications and other immune disorders (Baumgart and Sandborn, 2012). The management is medical and surgical, but treatment patterns have changed radically over the past years, with an increase in the use of immunomodulatory drugs and introduction of biologic therapy. Historically, up to 80% of CD patients had surgery at some stage (Cummings et al., 2008; Burisch et al., 2013). The biologic treatment is effective in achieving clinical response and remission in moderate-to-severe CD, but its impact on the rate of complications and, in particular, necessity of surgery is not clear (Sokol et al., 2014; Annese et al., 2016). A recent systematic review of clinical trials indicated that biologic treatment of CD reduces the rate of surgeries and hospitalizations compared with placebo (Mao et al., 2017). The design of the included trials (e.g., continuation of treatment despite minimal or no response) and *post hoc* feature of individual trial’s result limit the generalizability of the conclusion. On the other hand, the real-world data are heterogeneous and conflicting (Sokol et al., 2014; Annese et al., 2016). Some studies demonstrated that despite increasing intake of biologics, the rate of hospitalizations and surgeries has reminded unchanged or increased (Bewtra et al., 2007; Nguyen et al., 2007; Lazarev et al., 2010; Sokol et al., 2014; Jeuring et al., 2017). A meta-analysis of population-based studies indicated the reduction of risk for surgery in CD with time (Frolkis et al., 2013), but heterogeneity and other limitations (e.g., studies not designed to identify the specific cause) did not allow to assign a causal relationship of the trend with biologic treatment (Sokol et al., 2014). For example, the trend might have as well been explained by increase in the use of immunomodulators (Vester-Andersen et al., 2014) or by other factors (Jeuring et al., 2017).

It has been shown that the frequency of hospitalizations and surgical procedures among CD patients varies between countries (Odes et al., 2006; Vegh et al., 2015). Hence, the impact of biologic treatment on those events may also depend on the setting. In some countries, including Poland and United Kingdom, authorities limit the duration of biologic treatment to a maximum of 12 months. To our knowledge, our study is the first analysis of such a treatment scheme.

In the absence of reliable information on the consequences of biologic treatment of CD, there is limited ability to properly prioritize treatment, valuate new biologics, or determine their optimal sequence using a cost-utilitarian approach. As a result, most economic evaluations omitted or incorporated own assumptions regarding the impact of biologics on treatment patterns and rate of surgeries (Huoponen and Blom, 2015).

Therefore, we decided to conduct a study to assess real-world consequences of biologic treatment of CD in all adult patients with CD treated in Poland in the years from 2012 to 2014.

## Materials and Methods

### Study Design and Patients

This was a retrospective analysis of medical resource utilization among patients with CD treated with infliximab or adalimumab in the years from 2012 to 2014 in Poland. The cohort was identified from the database of the National Health Fund [in Polish, Narodowy Fundusz Zdrowia (NFZ)], a public payer for all medical services in Poland.

In Poland, biologic treatment was provided through a program called a Drug Program (i.e., a set of in- and out-patient services) that included separate procedures for acquisition of biologic agents, their administration or self-administration, and diagnostics during biologic treatment. The inclusion criteria for the program among adult patients were: (i) severe, active CD (activity index > 300) with lack of response to treatment, contraindications, or intolerance to treatment with steroids, immunomodulatory drugs, or other tumor necrosis factor-α inhibitor; or (ii) perianal fistulas which do not heal despite treatment with antibiotics and surgical treatment in combination with immunomodulatory therapy. The exclusion criteria were as follows: hypersensitivity; severe infections; moderate or severe heart failure; unstable angina; chronic respiratory, renal, or liver failure; demyelinating syndrome-like symptoms; alcoholism; active progressive liver disease; pregnancy or breastfeeding; the diagnosis of precancerous state and malignant tumors during the previous 5 years; and complications requiring a change of treatment (e.g., surgeries with the exception of procedures for closing fistulas). The induction treatment (around 100 days) was administered to all patients (with or without response). However, the maintenance treatment was available for the responders only. Overall, the biologic treatment was continuously administered for a period of no longer than 12 months. However, eligible patients could re-enter treatment, that is, start induction phase after 16 and 8 weeks since the last dose of infliximab and adalimumab, respectively.

The eligibility criteria for the study were at least one administration of biologics in the program for CD and age of 18 years or older during the first biologic treatment. Patients using infliximab or adalimumab in other programs and patients below 17 years of age at the first administration of biologics were excluded owing to different criteria for continuation of treatment.

### Data Source and Management

We used part of the database that initially contained information for all patients with inflammatory bowel disease in Poland and was created for the National Institute of Public Health – National Institute of Hygiene (Holko et al., 2018). The database contained information on date of birth, sex, province of residence (province), all in-patient and out-patient services and all medications, diet supplements, nutritional products, or medical devices on prescription. In this study, data on eligible patients were extracted. All medical resources used between the first and the last resource utilization in the years from 2012 to 2014 for each eligible patient were analyzed.

To verify the main hypothesis regarding the difference in resource utilization according to the exposure to biologic treatment, the observation period for each patient was divided according to an index date of the first administration of a biologic drug [before the first administration (pre-index period) and after the first administration of a biologic drug (post-index period)].

All medical procedures with the directional ICD-10 (the 10th revision of the *International Statistical Classification of Diseases and Related Health Problems*) code of CD or related intestinal or extraintestinal complications were considered as being directly related to CD. The medium, large, or complex surgical procedures [identified using the “diagnosis-related group” (DRG) codes] directly related to CD (as described above) and performed in any department of a medical center were considered as surgeries for CD (Supplementary Table S1). Hospitalization was defined as a planned or an unplanned hospital stay (including an emergency department) for more than 1 day.

The number of packs was used as a unit of antibiotics and systemic glucocorticoid intake.

The rates of surgeries for CD, hospitalizations for CD (excluding hospitalizations for surgical procedures), consumption of steroids, and consumption of antibiotics were the main endpoints of the study because they directly related to complications or worsening of CD. The rate of all resources (i.e., hospitalizations, ambulatory specialist consultations, or ambulatory services) irrespectively of the relation to CD and services related to administration of biologics and diagnostic procedures during biologic treatment were assessed to validate the resource selection process and to validate the conclusions against any confounders that were not directly observed.

The principal and secondary diagnosis ICD-10 codes established for each patient over the first 6 months of the study were used to calculate the Charlson comorbidity index score using the coding algorithm described by Quan et al. (2005). The Classification of Territorial Units for Statistics was used for grouping patients by the geographical regions of Poland.

### Statistical Analysis

All study outcomes and patients’ characteristics were analyzed descriptively and presented as a mean with *SD* or median with interquartile range (IQR) for continuous variables and as frequencies for categorical variables.

The generalized linear mixed models with Poisson or negative binominal (when overdispersion was present) distribution, log link, robust errors, period duration as a quantification of exposure, and random intercepts by patient were used to assess medical resource utilization during the study periods (pre- vs. post-index). The secondary analysis included the interaction of a variable describing study periods and that describing the quintile of total exposure to biologic treatment during the post-index period. All models included age, sex, comorbidity score, and region of Poland to control for possible confounders. The models with “exposure” variable allowed us to determine the incidence rate ratio (IRR) for each predictor while considering the differences in period duration. Model selection and assessment were based on the characteristics of dependent variable, residual distribution, random effect distribution, and Akaike information criterion. Average adjusted predictions were presented as adjusted means with confidence intervals (CIs) calculated using the delta method. Unless stated otherwise, the average adjusted marginal effects were indicated as adjusted difference in annualized rates.

Missing data were excluded from the descriptive analysis of an outcome, but patients with missing observations were included in mixed models. The Bonferroni correction for multiple hypothesis testing was incorporated. To ensure self-explanatory attribute of the results, the *p* values and CIs were adjusted with the correction, that is, the adjusted *p* values were presented as *p* values and CIs adjusted for multiplicity were presented as 95% CIs. The adjusted *p* value of less than 0.05 (nominal *p* value of less than 0.001) was considered statistically significant.

The study was reported in adherence with the Strengthening the Reporting of Observational Studies in Epidemiology Statement (von Elm et al., 2008).

Data preparation and statistical analyses were done using Access 2016 (Microsoft Co., Redmond, WA, United States) and STATA 14.2 (StataCorp, College Station, TX, United States). Figures were prepared using OriginPro 2017 (OriginLab, Northampton, MA, United States).

### Ethics Statement

This article does not contain any studies with animals or humans performed by any of the authors (retrospective database analysis).

## Results

### Characteristics of Patients

A total of 1613 patients were treated in the program for CD in the years from 2012 to 2014. Fourteen patients were excluded because of treatment with infliximab or adalimumab in other programs (psoriasis, ankylosing spondylitis, or ulcerative colitis) and 206 were excluded because of age.

The study included 626 patients treated with infliximab, 587 patients treated with adalimumab, and 180 patients treated with both biologics. A total of 1050, 285, 56, and 2 patients received one, two, three, and four biologic treatments, respectively, during follow-up. The mean age at the first administration of biologics was 31.9 years (*SD* 11.1; range: 17.0, 79.4), and 52.6% of the patients were male. Most of the patients (94.4%) had no life-threatening comorbidities (comorbidity score of 0), and 44.7% were from the eastern or central region of Poland. The rates of immunomodulatory drug and steroid use did not differ significantly between patients treated with adalimumab and those treated with infliximab at the index date and between patients during subsequent biologic treatments (**Table 1** and Supplementary Table S2).

**Table 1:** Characteristics of patients at the start of the first biologic treatment.

	Value
Number of patients	1393
Age in years at start of treatment, mean (*SD*)	31.9 (11.1)
Women, *n* (%)	661 (47.5)
Comorbidity score	
0, *n* (%)	1315 (94.4)
1+, *n* (%)	78 (5.6)
Region of Poland	
Eastern or central, *n* (%)	623 (44.7)
North or north-western, *n* (%)	409 (29.4)
South or south-western, *n* (%)	361 (25.9)
Biologic treatment	
Number of patients (%) with maintenance treatment^a^	1035 (74.3)
Total exposure in days, median (IQR)	314 (134, 365)
Rate of other medication use during biologic treatment	
Immunomodulators, *n* (%)	774 (55.6)
Steroids, *n* (%)	437 (31.4)

^a^*Patients continuing induction treatment at the end of the study period were excluded*.

The median observation period (pre- and post-index) was 1064 days (IQR: 1026, 1084), with a median post-index period of 644 days (IQR: 294, 994) and a median cumulative duration of biologic treatment of 314 days (IQR: 134, 365). Overall, the study included 1512.2 patient-years during the pre-index period and 2382.3 patient-years during the post-index period.

### Resource Utilization

Among the 678 patients, there were 1045 surgeries for CD, including 482 surgeries (346 patients) before, 150 surgeries (140 patients) during, and 413 surgeries (308 patients) after biologic treatment. Of 150 surgeries, 40.7% most probably resulted in discontinuation of biologic treatment, that is, they occurred during the estimated treatment period, between the last and the next expected administration of biologics according to the treatment schedule. The average rate of surgery for CD was reduced with biologic treatment by 27% in comparison to the pre-index period (95% CIs: 8%, 42%, i.e., -0.08 events per year, *p* = 0.001; Supplementary Table S3).

The rate of hospitalizations for CD was reduced by -0.73 events per year (*p* < 0.001). A significant effect of biologic treatment on the intake of steroids (-1.52 packs per year, *p* < 0.001) and antibiotics (-0.56, *p* < 0.001) was observed. The rates of all hospitalizations (adjusted difference of -0.40 events per year, *p* < 0.001) and all ambulatory consultations with a specialist (-2.93, *p* < 0.001) were significantly reduced, but the rate of all ambulatory services did not differ between the pre- and post-index periods (-0.02 events per year; 95% CIs: -0.24, 0.20). The reduction in the rate of all specialist consultation was partially compensated with additional services related to administration of biologics or performing diagnostic procedures during biologic treatment (**Table 2**).

**Table 2:** The annualized rates of medical services and medication intake before and after the first administration of biologics (pre- and post-index, respectively).

	Estimate	Pre-index	Post-index
Follow-up (patient-years)	Totals	1512.2	2382.3
Surgeries for CD^a^	Unadjusted mean (totals)	0.35 (482)	0.24 (563)
	Adjusted mean (95% CIs)^b^	0.32 (0.27, 0.38)	0.24 (0.20, 0.27)
	IRR (95% CIs)	–	0.73 (0.58, 0.92)^∗^
Hospitalizations for CD^a,c^	Unadjusted mean (totals)	1.53 (2306)	0.91 (2158)
	Adjusted mean (95% CIs)^b^	1.61 (1.43, 1.78)	0.88 (0.78, 0.98)
	IRR (95% CIs)	–	0.55 (0.47, 0.63)^∗∗^
Steroids (packs)	Unadjusted mean (totals)	4.39 (6635)	2.71 (6443)
	Adjusted mean (95% CIs)^b^	4.31 (3.60, 5.02)	2.79 (2.36, 3.23)
	IRR (95% CIs)	–	0.65 (0.52, 0.82)^∗∗^
Antibiotics (packs)	Unadjusted mean (totals)	1.75 (2639)	1.30 (3107)
	Adjusted mean (95% CIs) ^b^	1.81 (1.55, 2.08)	1.25 (1.08, 1.43)
	IRR (95% CIs)	–	0.69 (0.58, 0.82)^∗∗^
Services related to biologics^d^	Unadjusted mean (totals)	0 (0)	5.60 (13,291)
All hospitalizations^e^	Unadjusted mean (totals)	1.87 (2827)	1.51 (3587)
	Adjusted mean (95% CIs)^b^	1.90 (1.73, 2.08)	1.50 (1.36, 1.64)
	IRR (95% CIs)	–	0.79 (0.69, 0.89)^∗∗^
All ambulatory consultations with a specialist^f^	Unadjusted mean (totals)	6.06 (9160)	4.00 (9519)
	Adjusted mean (95% CIs)^b^	6.68 (6.04, 7.31)	3.75 (3.42, 4.09)
	IRR (95% CIs)	–	0.56 (0.51, 0.62)^∗∗^
All ambulatory services^g^	Unadjusted mean (totals)	0.56 (842)	0.47 (1110)
	Adjusted mean (95% CIs)^b^	0.53 (0.28, 0.78)	0.51 (0.31, 0.71)
	IRR (95% CIs)	–	0.96 (0.63, 1.45)

*CD, Crohn’s disease, IRR, incidence rate ratio. ^∗^*p* = 0.001; ^∗∗^*p* < 0.001. ^*a*^See Supplementary Table S1 for details. ^*b*^Average adjusted predictions of generalized linear mixed models with age, sex, region, comorbidity score, and binary variable indicating pre-index or post-index periods (Supplementary Table S3). ^*c*^Surgeries for CD and hospitalizations related to administration of biologics or performing diagnostics during biologic treatment were not included. ^*d*^Hospitalization, 1-day hospitalization, or outpatient visit indicated as for biologic administration or performing diagnostic procedures during biologic treatment. ^*e*^Surgeries for CD, hospitalizations for CD, hospitalizations related to biologic treatment, and hospitalizations not related to CD. ^*f*^All ambulatory consultations, i.e., consultations with simple diagnostic procedures (e.g., X-ray, ultrasound, urine, or blood tests), consultations solely for prescriptions, etc. ^*g*^Medical interventions performed as part of the “Outpatient Specialist Care” (in Polish,“Ambulatoryjna Opieka Specjalistyczna”; general examples: introduction of a catheter, biopsies, incision of abscess, and tooth extraction) were included*.

**FIGURE 1:**
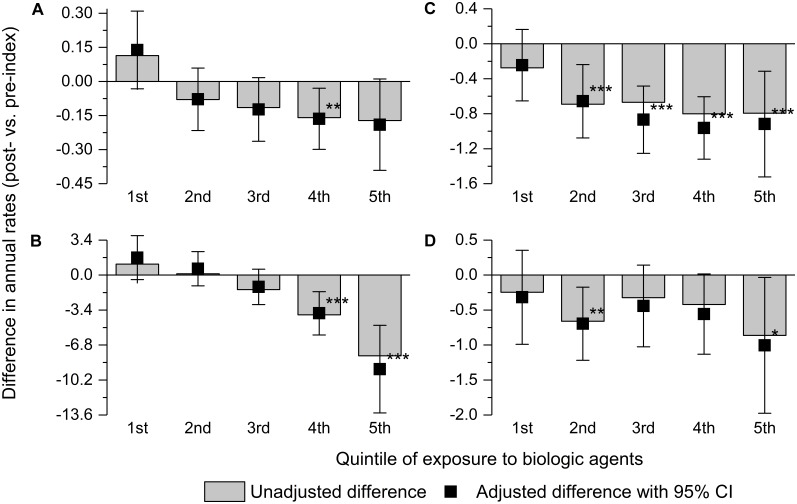
Adjusted and unadjusted difference between the post-index and pre-index periods in annualized rates of surgery for Crohn’s disease **(A)**, other hospitalizations for Crohn’s disease **(B)**, and consumption of steroids **(C)** and antibiotics **(D)** by the quintile of total exposure to biologic agents. Error bars indicate 95% confidence intervals. ^∗^0.05 > *p* ≤ 0.01; ^∗∗^0.01 > *p* ≤ 0.001; and ^∗∗∗^*p* < 0.001.

The reduction in the rates of surgeries, other hospitalizations for CD, and steroid intake increased with higher exposure to biologic agents. The trend was not observed for antibiotic intake (**Figure 1**).

## Discussion

The conflicting evidences on the impact of biologic treatment on the rate of complications and necessity of surgery in CD provided the rationale for this study. All adult patients with CD treated with infliximab and adalimumab in the years 2012–2014 in Poland were included in the study. Biologic treatment reduced the rates of surgeries, hospitalizations for other causes related to CD or its complications, and consumption of antibiotics and steroids in real-world setting.

The strengths of our study include the size and heterogeneity of participants, which was obtained by a non-selective inclusion of all patients treated with biologics in Poland. However, the source of the data constitutes an important limitation of the study. The data were obtained from an administrative database of medical resources and are susceptible to errors made during the reporting of the resources by providers (e.g., inaccurate ICD-10 codes). Moreover, the detailed information on clinical characteristics of patients’ disease was not available. However, only patients meeting the specific inclusion criteria (i.e., active CD – progression or relapse – during steroid or immunosuppressive therapy) were allowed to start biologic treatment in Poland and it is likely that all those patients from Poland were included in this study.

Although justified by the changes in Polish healthcare system in 2012 and incompleteness of data from 2015 at the time of data extraction, the duration of follow-up period limited to the years 2012–2014 is another drawback. The first biologic treatment in the study period was not necessarily the first ever biologic treatment in some percentage of the patients (the treatment was formally introduced in Poland in 2007). However, 52% of the patients were followed for at least 6 months prior to the first biologic treatment in this study. The duration of the follow-up period limited to 3 years was sufficient to analyze the impact of biologic treatment on the rate of the selected events, because the duration of biologic treatment was limited to 12 months in Poland and all the resources analyzed (including some types of surgeries) were allowed to be used during biologic treatment. Moreover, the relationship between biologic treatment and the reduction in the rate of surgeries and other resources was confirmed by assessing the association between that reduction and the level of exposure to biologic treatment.

Another limitation includes the assumption of the analyses, that is, the consumption of steroids and antibiotics was assumed to occur immediately after supply and all missing observations were treated as random. The comparison was affected by a potentially different course of the disease (e.g., diagnosis of steroid-refractory or steroid-dependent disease around the index date). Patients served as their own controls, which means that the possibility of a patient improving spontaneously at the start of biologic treatment as a result of the so-called “regression toward the mean” cannot be excluded. However, similar outcomes among all patients during the pre-index period and patients exposed to biologics for 100 days or less during the post-index period (most likely non-responders) suggest that the effect of this phenomenon is minimal.

Our study included patients with severe CD only, while most participants in clinical trials had moderate to severe CD. In addition to a different definition of the outcome and different settings, this explains the difference in the rate of surgeries between our study (0.24–0.35 per year) and clinical trials (0.06–0.13; Mao et al., 2017).

Several sensitivity analyses were conducted to address the limitations of our study (data not shown). The exclusion of patients with the pre-index period shorter than 6 months did not affect most of the conclusions. The assessment of consumption of steroids with daily dose as a unit instead of a pack indicated higher impact of biologic treatment (e.g., reduction of 62% vs. pre-index), but the models less accurately fitted the data owing to the semi-continuous character of dependent variable and required more complex methods. Another analysis showed similar outcomes among patients on combination therapy (i.e., administration of biologic agent with immunomodulatory drug for >50% of the period of biologic treatment) and those on monotherapy.

The study results are in line with the findings from some cohort studies and randomized clinical trials. In their meta-analysis of randomized clinical trials, Mao et al. (2017) showed that the odds for CD-related hospitalization and surgery are reduced with biologic treatment compared with placebo by 53% and 74%, respectively. Similarly, a meta-analysis of observational studies with various design (pre-treatment vs. post-treatment, responders vs. non-responders, etc.) indicated a reduction of 72% and 68% in the odds for hospitalization and surgery, respectively, with infliximab monotherapy (Costa et al., 2013), but some individual studies indicated an increase in the rate of surgeries with biologic treatment (Park et al., 2014). On the contrary, Lazarev et al. (2010), in a single-center study, showed no change in rate of surgery with increasing use of biologic treatment, and Jeuring et al. (2017) reported no association between reduction in the rate of surgeries and biologic treatment. Both studies focused on the total cohort of CD patients and did not assess management patterns at the patient level.

The level of reduction in the rate of surgeries and hospitalizations was lower in our study (27% and 45%, respectively) than that reported by Costa et al. (2013; 68% and 72%, respectively) or Mao et al. (2017; 74% and 53%, respectively). The observed differences may be explained by different settings, because it was shown that the frequency of hospitalizations and surgical procedures among patients with CD varies significantly between countries (Odes et al., 2006; Vegh et al., 2015). However, the observed correlation between the reduction in the resource utilization and the level of exposure to biologic agents suggested that the difference in the level of reduction in the rate of surgeries and hospitalizations between our study and others is mainly caused by the limitation of the treatment period to 12 months in Poland. The higher reduction of resources utilization in studies without this limitation suggested that it may be inadequate to achieve long-term clinical benefit from biologic treatment. On the other hand, it may suggest that biologic treatment prevents complications and worsening of CD in clinical practice, but mainly during treatment. Since January 2017, the biosimilar infliximab can be used in the treatment of CD for a longer period in Poland (up to 24 months). Possibly, future research regarding longer treatment period will be able to confirm these observations (i.e., the level in the reduction of surgeries depending on the allowed treatment period and/or the presence of the reduction during treatment only).

Limitation of the treatment period to 12 months in Poland, adverse events and loss of response preventing continuous long-term treatment, and different designs were presumably the reasons for the discrepancies between our study and population studies. The change of treatment patterns at the patient level cannot translate to the effect that can be observed in the whole CD population, especially if the rate of biologic treatment is low. The study showed that around half of the patients received biologic treatment in combination with immunomodulatory drugs and around half of them discontinued treatment before the end of the maximum allowed period of 12 months. Discontinuation of treatment may occur for several reasons, such as patient preference, achievement of stable remission, lack or loss of response, adverse events, or pregnancy. Even though the cause could not be determined, the high discontinuation rate indicates that there is an unmet treatment need in patients with CD. High relapse rates after discontinuation of treatment in this study (re-treatment in 26–40% of patients after a year) and in other studies identified in a systematic review by Gisbert et al. (2015) may indicate that biologic treatment requires continuous administration or setting long-term clinical goals.

The results of this study are clinically and economically relevant because the outcomes are considered to be markers of disease severity and contribute to the considerable share of healthcare costs of the disease. Furthermore, the results can be used to inform economic models created to assess the cost-effectiveness of biologics or to assess optimal treatment sequence. Finally, with the largest cohort of adult patients using biologic treatment to date (Costa et al., 2013; Annese et al., 2016) and a non-selective process of patient inclusion, the results of this study provide a sound argument in an ongoing debate on real-world outcomes of biologic treatment and changes in management patterns induced by the treatment.

## Data Availability

All data are presented in the manuscript or the supplementary materials. The dataset is available from the corresponding author upon reasonable request.

## Author Contributions

PH contributed to methodology, data management, analysis, validation, and visualization of the data and drafted the manuscript. PH and PK contributed to interpretation of the data. PK contributed to data acquisition. PK and AP contributed to project administration. All authors conceived and designed the study, contributed to editing the manuscript, and approved the final version submitted for publication.

## Conflict of Interest Statement

The authors declare that the research was conducted in the absence of any commercial or financial relationships that could be construed as a potential conflict of interest.
