# Supplementary material for: Impact of Biologic Treatment of Crohn’s Disease on the Rate of Surgeries and Other Healthcare Resources: An Analysis of a Nationwide Database From Poland
Source: Front Pharmacol. 2018 Jun 11;9:621. doi: 10.3389/fphar.2018.00621 (PMC6004509; doi:10.3389/fphar.2018.00621)
Supplement: Supplementary file 1 [file Table_1.PDF]

Supplementary Table 1. The "diagnosis related group" (DRG) and 10th revision of the *International Statistical Classification of Diseases and Related Health Problems* (ICD-10) codes used to identify Crohn's disease-related surgeries and Crohn's disease-related hospitalizations.

	**DRG codes**	**"Directional" ICD-10 codes**
Surgical procedures	F21 Comprehensive Small Intestine Procedures; F22 Large and Endoscopic Procedures of The Small Intestine; F31 Comprehensive Colon Procedures; F32 Large and Endoscopic Colon Procedures; F33 Medium and Endoscopic Colon Procedures; F34 Medium and Endoscopic Gastrointestinal Procedures; F42 Large Abdominal Surgery; F43 Medium and Endoscopic Treatment of Abdominal Cavity; F51 Comprehensive Treatments in Inflammatory Bowel Diseases; F52 Large and Endoscopic Procedures in Inflammatory Bowel Diseases; F53 Medium and Endoscopic Procedures in Inflammatory Bowel Diseases; F93 Medium Anal Treatments	K50; K50.0; K50.1; K50.8; K50.9; K51.0; K51.1; K51.3; K51.8; K51.9; K52.8; K52.9; K55.0; K55.2; K55.8; K55.9; K56.0; K56.1; K56.5; K56.6; K56.7; K57.1; K59.9; K60; K60.0; K60.1; K60.2; K60.3; K60.4; K60.5; K61.0; K61.1; K61.2; K62.4; K62.5; K62.8; K63.0; K63.1; K63.2; K63.8; K63.9; K65.0; K65.8; K65.9; K66.0; K66.8; K66.8; R10.1; R10.3; R10.4; R19.8
Other hospitalizations	Others (length of stay >1 day)	D50; D50.8; D50.9; D51; D51.8; D53; D63; E06.3; E40; E41; E43; E44; E44.0; E44.1; E46; E55.9; E58; E61.1; E64.0; K26; K26.5; K26.7; K26.9; K29; K29.0; K29.1; K29.3; K29.5; K29.6; K29.7; K29.8; K29.9; K30; K31; K31.8; K31.9; K50; K50.0; K50.1; K50.8; K50.9; K51; K51.0; K51.1; K51.2; K51.3; K51.8; K51.9; K52; K52.0; K52.2; K52.8; K52.9; K55; K55.0; K55.8; K56; K56.0; K56.1; K56.4; K56.5; K56.6; K56.7; K57; K57.5; K59.0; K59.1; K59.8; K59.9; K60; K60.0; K60.1; K60.2; K60.3; K60.4; K60.5; K61; K61.0; K61.1; K61.2; K61.3; K61.4; K62; K62.0; K62.4; K62.5; K62.8; K62.9; K63; K63.0; K63.1; K63.2; K63.3; K63.8; K63.9; K65; K65.0; K65.8; K65.9; K66; K66.0; K71.8; K73.2; K73.8; K90; K90.0; K90.2; K90.4; K90.8; K90.9; K91; K91.2; K91.3; K91.4; K91.8; K91.9; K92; K92.2; K92.8; K92.9; R10; R10.0; R10.1; R10.2; R10.3; R10.4; R11; R15; R19; R19.0; R19.8; R21; R22; R63.4; R68; R93.2
