# Supplementary material for: Impact of Biologic Treatment of Crohn’s Disease on the Rate of Surgeries and Other Healthcare Resources: An Analysis of a Nationwide Database From Poland
Source: Front Pharmacol. 2018 Jun 11;9:621. doi: 10.3389/fphar.2018.00621 (PMC6004509; doi:10.3389/fphar.2018.00621)
Supplement: Supplementary file 2 [file Table_2.PDF]

Supplementary Table 2. Patients' characteristics at start of treatment with infliximab (IFX) or adalimumab (ADA). Information on fourth treatment (2 patients) was omitted.

	**1st treatment**		**2nd treatment**		**3rd treatment**	
	**IFX**	**ADA**	**IFX**	**ADA**	**IFX**	**ADA**
Number of patients	734	659	149	194	23	35
Age in years at start of treatment, mean (SD)	31.6 (11.3)	32.3 (11.0)	31.3 (9.4)	31.5 (10.1)	34.8 (9.3)	31.1 (9.4)
Women, n (%)	348 (47.4)	313 (47.5)	63 (42.3)	98 (50.5)	11 (47.8)	17 (48.6)
**Comorbidity score**						
0, n (%)	688 (93.7)	627 (95.1)	144 (96.6)	185 (95.4)	21 (91.3)	34 (97.1)
1+, n (%)	46 (6.3)	32 (4.9)	5 (3.3)	9 (4.6)	2 (8.7)	1 (2.9)
**Region of Poland**						
Eastern or central, n (%)	355 (48.4)	268 (40.7)	79 (53.0)	86 (44.3)	13 (56.5)	18 (51.4)
North or north-western, n (%)	194 (26.4)	215 (32.6)	35 (23.5)	54 (27.8)	4 (17.4)	9 (25.7)
South or south-western, n (%)	185 (25.2)	176 (26.7)	35 (23.5)	54 (27.8)	6 (26.1)	8 (22.9)
**Biologic treatment**						
Number of patients (%) with maintenance treatment with biologics [a]	523 (78.4)	512 (81.0)	106 (77.4)	155 (89.1)	10 (58.8)	24 (80.0)
**Rate of other medication use during biologic treatment**						
Immunomodulators, n (%)	417 (56.8)	357 (54.2)	72 (48.3)	98 (50.5)	10 (43.5)	14 (40.0)
Steroids, n (%)	225 (30.7)	212 (32.2)	53 (35.6)	72 (37.1)	7 (30.4)	13 (37.1)
