# Supplementary material for: Impact of Biologic Treatment of Crohn’s Disease on the Rate of Surgeries and Other Healthcare Resources: An Analysis of a Nationwide Database From Poland
Source: Front Pharmacol. 2018 Jun 11;9:621. doi: 10.3389/fphar.2018.00621 (PMC6004509; doi:10.3389/fphar.2018.00621)
Supplement: Supplementary file 3 [file Table_3.PDF]

Supplementary Table 3. The statistical models for the assessment of medical resource utilization.

	**CD-related surgeries**	**CD-related hospitalizations**	**Steroids (packs)**	**Antibiotics (packs)**	**All hospitalizations**	**All ambulatory consultations**	**All ambulatory services**
**Fixed effects – IRR (95% CI)**							
Age (increase by 1 year)	0.99 (0.98, 1.005)	1.00 (0.99, 1.005)	1.00 (0.99, 1.01)	1.00 (0.99, 1.01)	1.00 (0.99, 1.003)	1.01 (1.005, 1.02)*	1.02 (0.99, 1.04)
Sex (female vs. male)	1.02 (0.80, 1.28)	1.07 (0.92, 1.25)	0.92 (0.76, 1.12)	1.17 (0.96, 1.42)	1.08 (0.94, 1.23)	1.43 (1.23, 1.67)	0.80 (0.48, 1.31)
Comorbidity index (1+ vs. 0)	1.27 (0.78, 2.07)	0.98 (0.69, 1.38)	1.12 (0.74, 1.68)	1.18 (0.78, 1.80)	1.06 (0.81, 1.39)	1.47 (1.14, 1.89)*	0.95 (0.35, 2.56)
North or north-western region (vs. eastern or central)	1.04 (0.80, 1.35)	0.90 (0.74, 1.09)	1.01 (0.81, 1.26)	1.22 (0.97, 1.53)	0.90 (0.76, 1.06)	1.10 (0.92, 1.32)	1.39 (0.78, 2.47)
South or south-western region (vs. eastern or central)	0.84 (0.62, 1.13)	0.96 (0.80, 1.16)	0.83 (0.65, 1.05)	1.44 (1.14, 1.83)*	1.07 (0.91, 1.25)	1.05 (0.87, 1.26)	1.78 (0.96, 3.27)
Post- vs. pre-index	0.73 (0.58, 0.92)*	0.55 (0.47, 0.63)*	0.65 (0.52, 0.82)*	0.69 (0.58, 0.82)*	0.79 (0.69, 0.89)*	0.56 (0.51, 0.62)*	0.96 (0.63, 1.45)
**Other parameters and model performance**							
Exp(intercept) (95% CI)	0.0009 (0.0006, 0.0014)*	0.004 (0.003, 0.005)*	0.012 (0.009, 0.0018)*	0.003 (0.002, 0.004)*	0.005 (0.004, 0.006)*	0.008 (0.006, 0.010)*	0.00022 (0.00008, 0.00063)*
Overdispersion parameter (95% CI)	-	-	2.75 (2.60, 2.95)	0.44 (0.05, 0.82)	-1.14 (-1.81, -0.47)	0.37 (0.09, 0.64)	1.06 (0.54, 1.57)
Random effect (95% CI)	0.32 (0.18, 0.58)	0.40 (0.31, 0.52)	0.23 (0.12, 0.47)	0.56 (0.39, 0.79)	0.27 (0.20, 0.37)	0.51 (0.42, 0.63)	2.22 (1.30, 3.81)
Akaike information criterion	4004.06	8462.56	13993.49	9329.95	9989.03	13891.90	4221.65
Root-mean-square error (events / packs)	0.61	1.16	8.60	2.30	1.61	2.99	2.47

All models were based on 2597 observations for 1393 patients (1 to 2 observations per patient) and included exposure variable indicating the length of a period. * $p < 0.05$.

IRR, incidence rate ratio.
